# A Multi-Layer LoRaWAN Infrastructure for Smart Waste Management

**DOI:** 10.3390/s21082600

**Published:** 2021-04-07

**Authors:** David Baldo, Alessandro Mecocci, Stefano Parrino, Giacomo Peruzzi, Alessandro Pozzebon

**Affiliations:** Department of Information Engineering and Mathematics, University of Siena, 53100 Siena, Italy; baldo@unisi.it (D.B.); alessandro.mecocci@unisi.it (A.M.); parrino2@unisi.it (S.P.); peruzzi@diism.unisi.it (G.P.)

**Keywords:** smart waste management, smart city, LoRaWAN, fire detection, smart bin, smart drop-off container, IoT, edge computing

## Abstract

Long Range Wide Area Network (LoRaWAN) has rapidly become one of the key enabling technologies for the development of Internet of Things (IoT) architectures. A wide range of different solutions relying on this communication technology can be found in the literature: nevertheless, the most part of these architectures focus on single task systems. Conversely, the aim of this paper is to present the architecture of a LoRaWAN infrastructure gathering under the same network different typologies of services within one of the most significant sub-systems of the Smart City ecosystem (i.e., the Smart Waste Management). The proposed architecture exploits the whole range of different LoRaWAN classes, integrating nodes of growing complexity according to the different functions. The lowest level of this architecture is occupied by smart bins that simply collect data about their status. Moving on to upper levels, smart drop-off containers allow the interaction with users as well as the implementation of asynchronous downlink queries. At the top level, Video Surveillance Units (VSUs) are provided with machine learning capabilities for the detection of the presence of fire nearby bins or drop-off containers, thus fully implementing the Edge Computing paradigm. The proposed network infrastructure and its subsystems have been tested in a laboratory and in the field. This study has enhanced the readiness level of the proposed technology to Technology Readiness Level (TRL) 3.

## 1. Introduction

Waste production has gradually increased with the development of both urban centres and consumer society. To render waste management systems efficient, it is important for the collection to keep the same pace as the generation. This enables municipalities to minimize the waste management costs and simultaneously facilitate recycling with separate collection schemes, which effectively reduce the environmental burden of waste (global warming, littering and so on). To this end, ad-hoc monitoring infrastructures may be set up for example to control filling level of public waste bins. However, such facilities could only give limited support if they would be employed per se since they lack of additional effective features (e.g., connectivity, video surveillance and distributed computing) so as to be able to collect a multitudinous amount of data on which Artificial Intelligence (AI) algorithms and models may be applied. In so doing, simple data (e.g., filling level of public waste bins) may acquire a much more valuable meaning whenever they are combined with assorted and considerably structured data (e.g., information retrieved by cameras). In so doing, precise estimates, forecasts and control may be ensured thus enabling preventing maintenance, prompt decision making, optimization of bins depletion procedures and management and widespread enforcing so as to fight and forestall acts of vandalism and service misuses.

For the purpose of solving the aforesaid problems, this paper illustrates an innovative and heterogeneous system so as to establish a smart waste management framework bearing in mind smart cities contexts. Indeed, it is based on a Low Power Wide Area Network (LPWAN) enabled by the Long Range (LoRa) Wide Area Network (LoRaWAN) protocol whose nodes are of a threefold species so as to satisfy as much tasks: the simpler ones are installed within public waste bins and they are in charge to diffusely monitor the relative filling level along with the inner temperature and whether the bins were overturned or not. The second ones are designed to be installed on drop-off containers: these nodes are more complex since they must manage a larger number of tasks, including the interaction with drop-off container users. The latter ones are embedded in Video Surveillance Units (VSUs) that are especially designed so as to guard public waste bins and drop-off containers. So as not to violate users privacy, VSUs do not perform neither images streaming nor images storing. Actually, captured images are locally processed, thanks to the computational capability VSUs are equipped with (and hence establishing an edge computing paradigm), and then only non-sensitive data are broadcast via LoRaWAN. Such information may be miscellaneous (e.g., number of people passing in front of the public waste bins, number of individuals that use the wrong bin hampering recycling, number of people who litter and so on): nevertheless, in the architecture proposed in this paper, image processing is employed to detect the presence of fires only and to generate alarms accordingly. Once that data coming from public waste bins, drop-off containers and VSUs are combined at the LoRaWAN network server layer, all this information can be fused to generate newer knowledge, extracting useful contents in order to achieve better predictions and management. In so doing, a modular, heterogeneous, widespread Internet of Things (IoT) system is set up. Summarizing, the objective is to design the architecture of a Smart Waste Management infrastructure which is grounded on a LoRaWAN network. As it was just introduced, this system goes beyond what similar works within the literature propose as the bulk of them deals with the problem just by tackling it from a single perspective only. In addition, this paper innovates the literature due to the concurrent achievement of the following objectives by the developed prototype:

- Measurement of the filling level of either public waste bins and drop-off containers;
- Detection of vandalism to both public waste bin and drop-off containers;
- Localization of drop-off containers;
- Users authentication;
- Video surveillance without harming people privacy;
- Establishment of an edge computing paradigm;
- Accomplishment of a higher level of data abstraction starting from simple information acquired by public waste bins, drop-off containers and VSUs;
- Arrangement of a LoRaWAN network including all the device classes the protocol addresses;
- Creation of an infrastructure serving as a potential backbone for all those facilities not relying on high bit-rates but needing of a pervasive monitoring network topology within the broad context of Smart Cities.

Moreover, while the architecture of the sensor nodes to be installed inside bins was partially described (exploiting only LoRa technology without implementing the LoRaWAN protocol though) in previous works [1,2], the architecture, as well as the operational principle exploiting customized LoRaWAN networking, of the other two types of nodes is at our knowledge novel in literature, together with the overall multi-purpose LoRaWAN architecture.

The rest of the paper is composed as follows. Section 2 reports related works within the literature, while Section 3 gives the detailed system overview. In Section 4 tests methodology and set up are introduced and the relative results are presented, and discussed in Section 5. Eventually, Section 6 highlights conclusions and final remarks.

## 2. Related Works

The most simple method to make waste collection more efficient is remote monitoring of the state of public waste bins. With real-time accurate data on the filling levels of public waste bins the most efficient collection vehicle route can be executed, avoiding superfluous emptying of bins that are insufficiently filled and also too late emptying of bins that are over-filled. The literature is plenty of related works trying to solve this issue in such a way and even the authors put forth their contribution in previous works [1,2]: the former introduced a prototype of low power smart public waste bin that was able to measure and remotely send, by means of a LoRa network, its filling level exploiting an off-the-shelf ultrasonic sensor; the latter improved the prototype that was previously shown by substituting the ultrasonic sensor with a waterproof one which was enclosed within a custom designed cap so as to refine its radiation lobe. Moreover, the literature pointed out the potential benefits of a smart waste management system either in smart cities contexts [3] and in professional and commercial environments [4].

Several studies suggested to monitor filling level by employing the same off-the-shelf ultrasonic sensor of [1]. In addition, some of these studies monitored the level of garbage by coupling the bins filling level and weight, as it is presented in [5], even though the measure of weight is not always reliable since different kind of wastes have different specific weight. Along with filling level, also inner temperature and ratio of carbon dioxide to oxygen may be sampled and remotely forwarded by means of cellular modules, with the aim of optimizing waste collection by resorting to the ant colony algorithm and, at the same time, to forecast garbage level by leveraging on data mining approaches [6]. Carbon dioxide is not the only gas which may exhale form public waste bins. Indeed, garbage tends to produce smelly gases as long as it remains within bins. To this end, Misra et al. [7] developed a system to monitor such a phenomenon together with bins status. Moreover, biodegradable wastes are prone to produce methane [8] which is a hazardous gas. Hence, in [9] an IoT system based on smart bins that are capable of sensing such a phenomenon along with other quantities (e.g., the filling level) was realized. Cellular technologies, in particular Global System of Mobile Communications (GSM) and General Packet Radio Service (GPRS), were also exploited in [10,11]: in the former smart public waste bins send Short Message Service (SMS) notices containing their filling level, while in the latter bins complete status is transmitted by exploiting the aforesaid cellular technologies. In the same vein, also [12,13] took advantage of SMSs to notify bin filling. However, communication over GSM/GPRS entails pretty high running costs though and hence it is far preferable to move towards other technologies. Enabling smart bins with WiFi, as it is shown in [14,15,16], could be a remedy but, as a drawback, coverage needs to be widely ensured.

Even though off-the-shelf ultrasonic sensors may perform quite well, especially for prototypical stages, they are characterized by two major downsides: most of the times they are not waterproof and they have too wide radiation lobes to be employed in any situation. This was the main reason why the authors investigated the solution proposed in [2]. In the same way, also [17,18] adopted the same ultrasonic sensor: the former in order to perform a smart waste bin, while the latter so as to realize a water level monitoring system. However, there exists other kind of finer ultrasonic sensors which may be compared with the one of [2]. An instance is the one reported in [19] where one of those is utilized to transform a public waste bin in a smart one so as to accomplish a smart waste management system which also accounts for algorithms for the depletion procedure planning.

Albeit ultrasonic sensors are the most suitable and robust ones whenever the operating scenario is dusty and subject to debris, residues and filth (e.g., within public waste bins), even infrared sensors were taken into account in spite of the fact that their performances could be hindered by operating circumstances. For instance, such sensors were exploited in [20,21]: unfortunately, though, since they were placed at the bin upper rim, they could only detect the moment in which the bin gets full rather than its filling level.

One of the targets a smart waste management system should aim at is the incentivization of recycling. Such a task becomes even tougher to achieve in massively crowded place. Possible solutions are mechanical recovery of the collected waste or in-line sorting within the bin. Such a system was described in [22] where Makkah and holy sites were considered as case study. Another feature that smart waste management could offer, could be to assess the fees for that service based on its use. This could be accomplished by embedding a Radio Frequency Identification (RFID) reader in public waste bins, along with all those components that make them smart, while users should be given with RFID tags. In so doing, wastes could be only thrown provided that users identified themselves beforehand. This idea was developed in the study published in [23].

Waste management may also include the control of wastewater tanks and basins. To this end, Ref. [24] devised a virtual sensor networks whose nodes are drones which are in charge to sample water coming from those basins by means of an especially developed probe. Waste management quality of service may be also enriched by taking advantage of VSUs along with the deployment of smart public waste bins. Indeed, data coming from cameras and data coming from bins may be collected and jointly analyzed so as to notify whenever garbage fills up bins [25]. Sadly, though, whenever cameras are employed within smart cities, privacy issues may easily arise [26]. Hence, images should be processed in advance aiming at extracting meaning features so not to violate inhabitants privacy: that was the basic idea standing behind the way VSUs work in this paper. Thus, on the one hand people privacy remains unharmed and, on the other hand, an edge computing paradigm is implicitly set up thanks to computational capability of VSUs.

Nowadays, edge and fog computing are increasingly gaining momentum with respect to cloud computing. Indeed, while some time ago huge amounts of data were centrally processed at a cloud level thus increasing network traffic and latency, in recent years such a computational burden has been decentralized thus taking place at terminal nodes of the network (i.e., edge computing) or at a distributed widespread level of the network (i.e., fog computing), thus leaving the cloud for data storage and just few light computing operations. In so doing, only valuable data, or at least compact formats of the original, will flow through the network and, as a direct consequence, both traffic and latency would be reduced. Some surveys shed light on the characteristics (e.g., delays, costs and capabilities) of fog computing in comparison with cloud computing [27], while other works thoroughly reviewed the paradigm concepts, standards, emerging trends, open issues, future challenges and application scenarios thus underlying the computing scheme ductility [28]: from big data analytic to computational offloading, from farm applications to distributed content delivery and caching up to smart cities applications. For what concerns the latter ones, two very well written and comprehensive surveys gave valuable insights [29,30], while [31] lingers over the application of fog computing within all those smart fields that are enclosed within the framework of smart cities (e.g., smart healthcare, smart parking and smart waste management). Fog computing may be also exploited in smart waste management systems in order to minimize the path during bins depletion by constructing time variant graphs whose nodes only account for full bins and then, at a fog layer, the shortest path problem could be solved [32].

The potentialities of adopting edge computing paradigms in a broad sense were pointed out [33], while the benefits in the ambit of smart cities frameworks were widely discussed in the survey [34]. Applying edge computing in LPWAN (e.g., LoRa based ones) whose nodes have limited computational capabilities is challenging but there exist studies which proved the feasibility and indicated the benefits [35]. Edge computing applied to smart bins and cameras may also aid in encouraging users in accomplishing recycling thus avoiding fines. Such an idea was implemented in [36] where an edge computing architecture which is able of detecting waste disposal violation in real time was developed by establishing a system very similar with the one that will be presented later on in Section 3. Apart from waste management, edge computing could generally find a much broader employment within smart cities where multiple facilities (e.g., public lighting, parking structures, energy consumption in buildings, waste management, carpooling, tourism and taxi cabs) may be created, run, maintained and enhanced. This was the scope that motivated [37,38]: the former dealt with the problem by leveraging on Single Board Computers (SBCs), while the latter performed the aforesaid tasks by setting up a comprehensive infrastructure which was developed and tested in the city of Messina, Italy.

A strategy in order to make smart waste management systems even more smarter could be to rig them with AI algorithms and machine learning models. For instance, bins filling level may be forecast, by means of AI, on the basis of historical data [39]. In addition, AI may be useful in order to produce real time waste related information by means of both descriptive and predictive data analysis approaches [40]: the former provides information associated, for example, to waste amounts by distinguishing either on locations and seasons by elaborating dataset of feature; the latter yields predictions by relying on time series. Earlier on it was stated that performing recycling at a later stage (i.e., once that users have already thrown garbage) could be pretty effective. To this end, AI could definitely help out by resorting to computer vision algorithms in order to automatically sort trash within bins [41]. Computer vision is not the only way in which AI may be applied within smart waste management systems: Convolutional Neural Networks (CNNs) [42,43,44], decision forest regression models [45] or random forest classifiers [46] are some other examples. Filling level of public waste bins may be also estimated by making use of logistic regression models set up over machine learning techniques [47] or combined with graph theory [48]: these are other methods so as to optimize routes during depletion procedures. In addition, such an optimization problem may be also worked out by adopting deep neuroevolutionary techniques so as to build up recurrent neural networks predicting the waste generation in a robust fashion (i.e., by taking into account uncertainty) [49] or even by employing data analytics platforms [50].

There exist also other solutions which may be compared with the one of this paper. For instance, the context of exploiting heterogeneous data for a smart waste management system was put forth in [4], where filling levels of smart drop-off containers spread out across Luxembourg were combined with GPS information from tracking devices installed on collection trucks. However, drop-off containers were provided with connectivity by resorting to Sigfox technology, and no VSUs were implemented. Similarly, Ref. [51] resembles smart public waste bins that will be introduced later on since it is able to sense bin filling level and to transmit such information via a wireless mesh network. In addition, similar duty-cycling policies are worked out in order to save power. However, connectivity performances cannot be compared to the ones of this paper since the mesh network [51] relies on is built over a technology comparable to WiFi. Similarly, Ref. [52] developed a system like [51] relying on a GSM connectivity for each of the bins. However, such a strategy entails massive running costs, while LoRaWAN links allow similar connectivity without this burden. In the same fashion, Ref. [53] proposes a smart bin monitoring system notifying alerts on its full state via SMS thus implying the same associated shortcomings. In contrast, Ref. [54] proposed a waste management system to be deployed in rural environments whose nodes were enabled by LoRaWAN capabilities. Such nodes can be compared to the smart drop-off containers of this paper (that will be described later on) albeit the former ones also sample their weight along with their temperature and their filling level. In so doing, waste collection routes may be dynamically optimized thus decreasing associated costs. Concerning users authentication, Ref. [23] developed a similar device performing authentication via RFID readers on bins and providing users with RFID cards. The literature also proposes systems for smart waste management running on renewable sources of energy like the smart drop-off containers of this paper. For instance, Ref. [55] exploits solar energy for powering a smart bin. Regarding connectivity and network infrastructure architecture, the work of this paper can be compared with [56] where similar sensors, even a GPS, are embedded in smart bins enabled with LoRa connectivity: the latter ones send data to a gateway that forwards the information towards a remote server via Message Queuing Telemetry Transport (MQTT).

On the other hand, there exist also commercial solutions that can be compared with the one of this paper. Bigbelly [57] is a solar-powered rubbish-compacting bin that can be installed in the most diverse spots within the city (e.g., public spaces, university campuses, parks and so on) thus implementing a de-facto smart waste management system through an ad-hoc IoT network spread in over 50 countries. Similarly, Contelligent [58] provides customers with both smart bins and a live web platform reporting the status of the deployed bins so as to have a punctual knowledge of their status. Bine [59] fuses IoT and big data contexts with the view of improving the management of public wastes. Indeed, Bine bins are able to recognize, sort and compact wastes while monitoring the bin filling level by wirelessly sending such information to a remote processing system. Ecobins [60] devised smart bins capable of monitoring and forwarding their filling level along with allowing user authentication like the system proposed in this paper. In addition, all of the gathered information is made available via a mobile web application. Similarly, Ecomobile [61] designed smart bins capable of compacting trash. Commercial solutions may also be able to provide a full facility for waste management like Nordsense [62] that by means of smart drop-off containers performs garbage trucks fleet management by evaluating an intelligent routing scheme so as to optimize depletion procedures.

In conclusion, to the best of our knowledge, neither commercial solutions nor state-of-the-art proposals solve this issue by employing a heterogeneous, multi-layer smart waste management system including smart bins, smart drop-off containers and VSUs that are part of a city-scaled LoRaWAN network thus adopting and implementing edge computing paradigms.

## 3. System Overview

**Figure 1:**
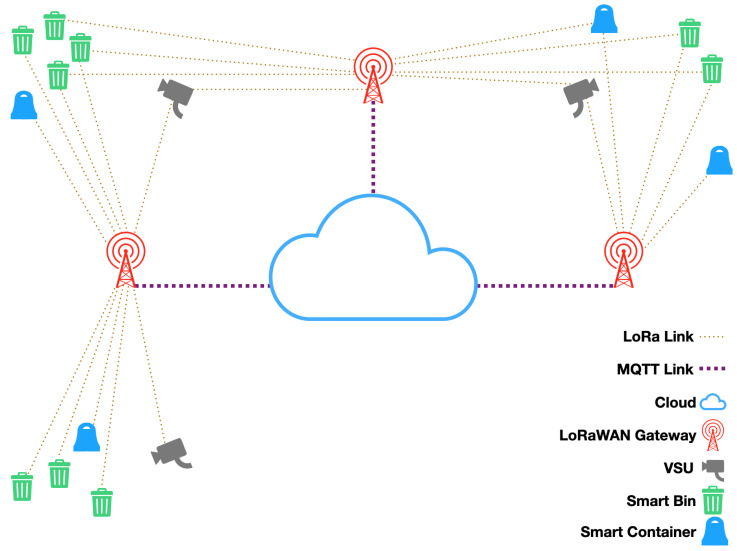
Smart waste management system overview.

The block diagram of the aforesaid smart waste management system is depicted in Figure 1. It makes use of a multi-layer LoRaWAN network whose nodes are of three different typologies, namely smart bins, smart drop-off containers and VSUs. All of them wirelessly transmit data to any LoRaWAN gateway in their closeness by exploiting LoRa links by establishing a frequency diversity scheme via a frequency hopping technique amid 8 different channels belonging to the 863÷870 MHz Industrial, Scientific and Medical (ISM) band and abiding by the regional regulation concerning the time occupancy of ISM bands [63] (i.e., in Europe and for the aforesaid ISM band, the spectrum can be occupied for no longer than the 1% of the time by each of the transmitters).

The three types of nodes host a firmware implementing a different typology LoRaWAN device, according to the various functions the nodes are devoted to. According to the LoRaWAN specification, three typologies of devices are envisaged:

- Class A devices (where A stands for all) are characterized by the lowest power consumption since downlinks are allowed only in two short receive windows after any uplink. This class is expected to be employed for end nodes in charge of simple data collection;
- Class B devices (where B stands for beacon) have scheduled extra receive slots in addition to the Class A random ones. So, the purpose of Class B is to have end-devices available for reception at a predictable time, in addition to the reception windows that follows the random uplink transmission from the end-devices of Class A. This entails for a possible remote control of some activities, at the price however of a higher power consumption;
- Class C devices (where C stands for continuous) includes end-devices, also known as Gateways, with maximal (i.e., nearly continuously open) receive slots within which they are capable to simultaneously handle packets incoming onto multiple frequencies. Such devices are obviously characterized by a large power consumption and thus are usually mains powered.

Such a multi-layer structure is reflected in the proposed system architecture which is composed of different types of end nodes of growing complexity, thus implementing all these three classes:

- Smart bins are the simplest devices in the network. They are in charge of synchronously transmitting a set of data collected by sensors deployed inside public waste bins. Since no downlinks are required, these are LoRaWAN Class A devices, battery-powered and provided with duty-cycling routines to reduce at minimum the power consumption. Architecture of smart bins is presented in detail in Section 3.1;
- Smart drop-off containers are characterized by a larger computational load. Besides implementing the same data acquisition functions of smart bins, they are also in charge of identifying drop-off container users, allowing waste delivery by unlocking the drop-off container cover. Since the functioning of these devices is more power consuming, they are powered by an energy harvesting system based on Photo-Voltaic (PV) panels. Such a larger power availability fosters the implementation of a LoRaWAN Class B device which allows to set up asynchronous downlink requests. The structure of smart drop-off containers is described in Section 3.2;
- VSUs are obviously the more power hungry devices in the network since they have to provide the largest computational load and they must include a camera whose power consumption is by far larger than the one of any other component of the system. According to the different operation mode, VSUs can implement either a LoRaWAN Class A or Class B device. The two different operation modes, as well as the selected class for each mode, will be described in Section 3.3.

All these devices communicate with the LoRaWAN server through a set of Gateways which implement LoRaWAN Class C devices. In particular, all the end-nodes encrypt the packet payload twice by making use of either the Network Session Key (NwkSKey) and the Application Session Key (AppSKey) by means of an Advanced Encryption Standard (AES) (i.e., AES-128). Moreover, all the classes of nodes transmit employing a power output of 14 dBm (i.e., the maximum allowed one by the regulations [63]), a Spreading Factor (SF) of 7 since it ensures the needed coverage requiring the minimum amount of energy to broadcast, a bandwidth of 125 kHz and a Coding Rate (CR) of 4/5. Even though lower CRs augment the probability of successful communications, the minimum CR was selected so as to minimize the power consumption during transmissions. The Gateways forward such information to the cloud by exploiting the Message Queuing Telemetry Transport (MQTT) protocol. The cloud hosts a LoRaWAN network server and a LoRaWAN application server. Every component of the system will be thoroughly shown in the following subsections.

The system presented in this paper covers the layers composing smart waste management solutions identified according to the literature [64,65]: perception layer, network layer, middleware layer, application layer and business layer. Perception layer includes both smart bins, smart drop-off containers and VSUs since they are in charge of perceiving all the phenomena which are of interest for the smart waste management system beforehand introduced (i.e., filling level of either bins and drop-off containers, identification of vandalisms, users authentication, drop-off containers position and fire recognition). The network layer is represented by the whole LoRaWAN infrastructure. In particular, the fact that all of the device classes foreseen by the protocol are included in the network represents a valuable novelty, since the bulk of works within the literature showing LoRaWAN networks only account for Class A and Class C devices. Moreover, the long link connectivity provided by LoRaWAN [66,67,68] allows for a considerable constraint relaxing related to the position of the entities falling within the perception layer. The middleware and the application layers are respectively identified in the LoRaWAN network server as well as in the LoRaWAN application server: both of them play a key role since they assure data management, storing and showing. Finally, the business layer is completely borne by the municipal companies availing the smart waste management system.

### 3.1. Smart Bins

**Figure 2:**
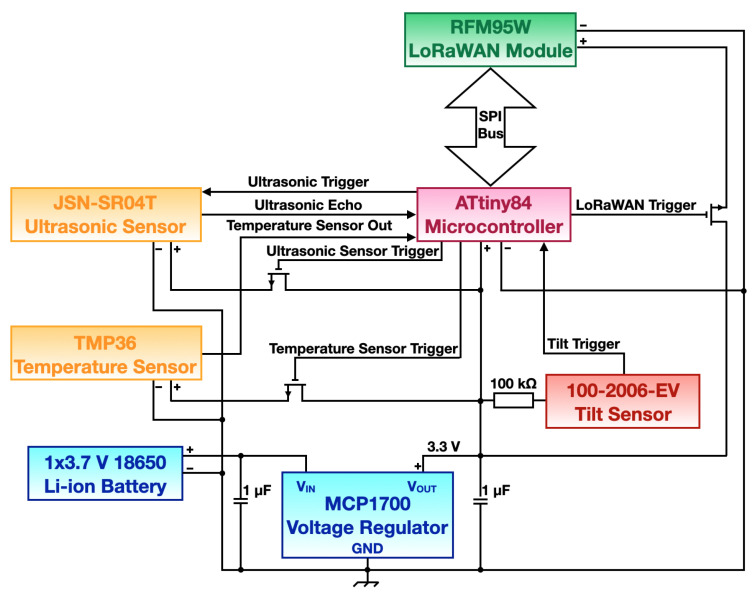
Smart bin block diagram.

The block diagram of a smart bin is reported in Figure 2: it is conceived so as to allow for a modular structure in order to only turn on the single components whenever they are needed and then to immediately turn them off achieving a limited consumption thus extending the battery lifetime.

#### 3.1.1. Components Description

As it has been just said, smart bins were designed so as to minimize power consumption. Indeed, they are battery powered without resorting to any energy harvesting technique. Such a choice could seem to be a naive solution at a first glance. However, since they are supposed to be deployed in an ample number across the environment which could be strongly heterogeneous (i.e., in some locations there could be sources of renewable energy while in some other spots it could not be so), avoiding the employment of energy harvesting modules in favour of an energy efficient management was preferred. In so doing, also production costs may be reduced due to the fact that a series assembly of smart bins may be commissioned. Hence, a 3.7 V 18650 Li-ion rechargeable battery providing 3500 mAh was chosen to power the bins. Since the components of the circuit need to operate at 3.3 V, a voltage regulator was required: hence, the MCP1770 low quiescent current low dropout voltage regulator [69], produced by Microchip, was adopted. As its datasheet suggests, two 1 μF capacitors were respectively introduced at the input and at the output so as to filter out noise and stabilize output voltage.

The core component of the smart bin is its high performance low power 8-bit microcontroller: the ATtiny84 produced by Atmel [70]. The reasons why this microcontroller was employed mainly are its ease of use, its limited power consumption and its low cost. Its firmware implements an energy saving policy which achieves an overall restrained energy consumption for the entire sensor node. Both the things (i.e., the policy and the battery lifetime estimation) will be described later on.

Smart bin sensors can be divided into two categories according to the way they are sampled by the microcontroller: synchronous (i.e., the ultrasonic and the temperature sensors) and asynchronous (i.e., the tilt sensor). The former ones are read by the microcontroller at every cycle of its firmware (i.e., on a time basis), while the latter generates triggers that the microcontroller deals as interrupts. With the aim at diminishing energy requirements, synchronous sensors are respectively enabled by the microcontroller only when their measurements are needed and only for the minimum required time. This task is performed by employing two N-type MOSFETs, which are driven by the microcontroller, that switch on and off each of the synchronous sensors. The ultrasonic sensor is a waterproof one, so as to withstand bin cleaning procedures, and it is exploited to measure the filling level of the bin. It is the JSN-SR04T [71], that is produced by Jahan Kit Electronic, which is the same employed in [2]: this sensor operates at a frequency of 40 kHz, featuring a distance detection range of 20÷600 cm. Its functioning is extremely simple: once that it is triggered by the microcontroller, the sensor emits ultrasound waves that are reflected back whenever they meet obstacles (e.g., garbage in the public waste bins) and at the moment in which they come back to the sensor, it notices such event to the microcontroller via the echo signal. The microcontroller is in charge of measuring the Time of Flight (ToF) (i.e., the elapsed time between the trigger and the echo) so as to evaluate the distance *d* according to the following equation:

(1) $$d=\frac{v_{s}\cdot t_{ToF}}{2}$$

where $v_{s}$ is the speed of sound and $t_{ToF}$ is the ToF. As it was shown in [2], the sensor is housed within a custom designed cap so as to refine its radiation lobe. The temperature sensor is the TMP36 [72] produced by Analog Devices. It is an analog sensor, therefore the microcontroller samples it via its embedded Analog to Digital Converter (ADC), it has a scale factor of 10 mV/°C and a linear behavior in the range −40 °C ÷ 125 °C providing 750 mV at 25 °C. In other words, the measured temperature *T* can be evaluated resorting to the equation

(2) $$T=\frac{V_{out}-750}{10}+25$$

where $V_{out}$ is the sensor output voltage expressed in mV. The tilt sensor is exploited to sense whenever the bin is overturned. Most of the times it is due to vandalism and such events need to be monitored and notified as soon as possible so that an operator could act. To this end, the 100-2006-EV tilt sensor [73], produced by Mountain Switch, was selected since it is a passive sensor behaving as a switch closing whenever the bin is overturned of at least 30°. In such a case, the sensor generates an interrupt which is managed by the microcontroller that signals the overturning event. This implies that the sensor has no consumption at all when it is not activated and a negligible one when it acts as a short circuit due to the 100 kΩ series resistor.

Connectivity is ensured by the RFM95W [74], produced by HopeRF, LoRaWAN module which embeds an SX1276 [75] LoRa chip produced by Semtech. It is driven by the microcontroller via the Serial Peripheral Interface (SPI) protocol. The module is enabled by following the same fashion of the one of the sensors: the microcontroller switches the module on via an N-type MOSFET just before the transmission and then it shuts it as soon as the communication is over. A rough estimation of the overall cost of the device can be made taking into account the prices of the single components, assuming that with mass production such a cost may notably decrease. However, the prototype presented in this section has an overall cost of ∼42 €. This value is notably lower than the one of the single bin, that is in the order of some hundreds of euros: since bins may be subject to vandalisms and deterioration due to their intense usage, the low cost of the electronics is a mandatory requirement in order to foster the adoption of such a technology.

#### 3.1.2. Energy Saving Policy and Battery Lifetime Estimation

The energy saving policy smart bins act is implicitly accomplished by the routines that are coded in the firmware running on the microcontroller. The target of the policy is to solely perform the strictly needed operations by activating the components of the node for the minimum required time and deactivating them as soon as their relative tasks are over. The firmware performs sensor measurements and data sending every half an hour (i.e., $t_{c}=1800$ s). Such a sampling frequency is enough to monitor data whose variation is extremely slow. Hereinafter, the policy implemented by the microcontroller is described step by step:

1. It enables the temperature sensor (by means of the dedicated MOSFET) and it acquires and stores 10 samples. Then it disables the sensor (still exploiting the same MOSFET) and it averages the samples. Finally, it stores the mean temperature;
2. It switches on the ultrasonic sensor (via the dedicated MOSFET) and, similarly as it was done with the temperature sensor, it performs and saves 10 measurements. After that, it switches off the sensor (by making use of the same MOSFET) and averages the measurements. Eventually, it saves the mean distance which represents the mean bin filling level;
3. It sorts out all the data coming from the sensors in a payload, whose structure is described below, so that it can be transmitted via LoRaWAN;
4. It turns on the LoRaWAN module (by controlling the dedicated MOSFET) and it arranges the transmission by means of the SPI bus. As soon as the broadcasting is over, the microcontroller turns off the communication module (by acting on the same MOSFET);
5. Finally, it enters in a sleep mode so as to save energy. It remains in such a state until the next samples are required (i.e., when half an hour has been passed since the current firmware cycle was started). The microcontroller awakes from the sleep mode thanks to the overflowing of the internal Watchdog Timer (WDT).

Earlier on, it was said that the tilt sensor behaves in an asynchronous way. Indeed, it is not involved within the routine explained above. Actually, it is managed by the microcontroller as an additional interrupt source along with the overflowing of the WDT: whenever the bin is overturned, it acts as a short circuit firing an interrupt to the microcontroller. The latter may handle such a signal in a twofold manner: if it is in sleep mode, it immediately awakes keeping track of the instance of the event (i.e., bin overturning) and by starting the routing from point 1; if it is already operating (i.e., from point 1 to point 4 of the routine), it handles the interrupt by recording the occurrence of the same event.

The LoRaWAN payload contains the mean temperature and the mean filling level as well as the overturning state of the bin. Each of those quantities are coded in a byte: hence, smart bins transmit 3 B length LoRaWAN packets. In so doing, each of the packet has a Time on Air (ToA), which can be evaluated by resorting to the formulas reported either in RFM95W datasheet [74] and in the one of SX1276 [75], of $t_{ToA}=51.46$ ms.

Besides ToA and in order to perform an estimate of the node lifetime, also the functioning period of all of the sensor node components along with their current consumption are needed. For what concerns the periods, they will be considered by referring to a single firmware cycle. Regarding current draws, they were measured by exploiting a Fluke 179 true RMS digital multimeter [76]. Either the functioning periods and the current draws that will be listed below were assessed on an average basis. The only components which are constantly activated are the voltage regulator and the microcontroller. The former has a functioning period equal to $t_{c}$ during which it draws 1.6 μA. The temperature sensor takes 20 ms to set up and to acquire 10 samples during which it requires 50 μA. The ultrasonic sensor employs 3 s to set up and to perform 10 distance measurements needing 8 mA. The LoRaWAN module is the energy hungriest component since it necessitates of 90 mA to set up and to transmit by taking 70 ms (it is worth noticing that such time is greater than ToA since the module requires to set itself up before transmitting). Finally, the microcontroller operates at the frequency of 1 MHz by relying on its internal oscillator so as to minimize its power consumption. Moreover, albeit it continually runs, it switches its functioning mode amid the active one and the sleep one thus entailing different consumption. During active mode it draws 490 μA for a period of 5 s. Such a time is necessary to accomplish the steps from point 1 to point 4 of the aforementioned routine. On the other hand, the microcontroller requires 4.6 μA while it is in sleep mode for keeping on incrementing the WDT. Such a functioning state lasts for a period of time of 1795 s provided that no interrupts from the tilt sensor are experienced. For summarizing purposes, all the functioning periods and the current draws are reported in Table 1 for each of the smart bin components.

At this stage, an estimate of the battery lifetime can be assessed. Passive components as well as capacitors and MOSFETs could be considered having a negligible contribution to this estimate. Moreover, the role of the tilt sensor is neglected as well since it may fire the routine restarting at random thus it cannot be estimated in a rigorous way. The estimation method relies on the data in Table 1. The overall node current absorption for each cycle of the firmware ($c_{c}$) can be evaluated as:

(3) $$c_{c}=\frac{1}{t_{c}}\sum_{i=1}^{6}c_{i}t_{i}≃0.0244\;\mathrm{mA}.$$

In order to ease the procedure, the node hourly absorption ($c_{h}$) can be evaluated:

(4) $$c_{h}=\frac{3600c_{c}}{t_{c}}=0.0488\;\mathrm{mA}.$$

Therefore, since the Li-ion battery powering the node has a capacity of 3500 mAh, the estimate of the node lifetime $\hat{l}$ can be computed as:

(5) $$\hat{l}=\frac{3500}{c_{h}}≃71,721\;h$$

which approximately corresponds to 2988 days. Despite this is only a rough estimate of the node lifetime, it confirms the effectiveness of the energy saving policy and the optimal low power capabilities of the smart bin building blocks.

### 3.2. Smart Drop-Off Containers

**Figure 3:**
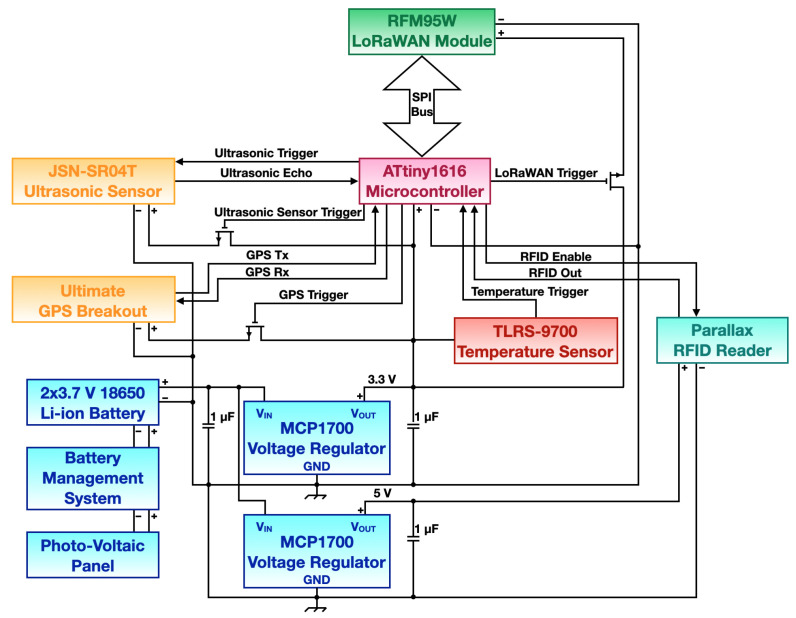
Smart drop-off container block diagram.

Smart drop-off containers share some of the features of smart bins, even if additional capability were added: these include an RFID reader for user authentication and a GPS module for the drop-off containers localization, since they often get moved in different spots. Similarly, smart drop-off containers are provided with an energy harvesting solution based on the use of PV panels. A block diagram of a smart drop-off container is shown in Figure 3.

#### 3.2.1. Components Description

Similarly as for smart bins, the architecture of smart drop-off containers aims at reducing the overall power consumption adopting low power off-the-shelf components. Nevertheless, some of the components included in this device require either to be always on (i.e., RFID reader) or are characterized by inherently high power consumption (i.e., GPS module): this means that the whole system is not able to rely only on batteries for long periods. Smart drop-off containers are then provided with an energy harvesting solution that will be described in detail later on.

The core of the smart drop-off container is represented by an ATtiny1604 microcontroller by Atmel: this component was chosen in place of the ATtiny84, used for smart bins, since it presents a key feature that is required for the realization of the hardware structure of the system. Indeed, it is provided with a 16 kB flash memory which is necessary since some of the components require cumbersome libraries for their functioning. At the same time, the ATtiny1604 microcontroller presents the same number of pins as the ATtiny84 (i.e., 14 pins) and a limited and comparable power consumption. All of the relevant features of the ATtiny1604 microcontroller are listed in Table 2.

The system features then, similarly to the smart bin, the JSN-SR04T ultrasonic sensors for trash level detection: the characteristics of this sensor were described in Section 3.1.1. Concerning temperature, a different sensor is employed: indeed, the component integrated in the smart drop-off container is a TLRS-9700 thermal cutoff device behaving as a switch when the temperature overcomes a certain threshold. In the proposed context, this devices is employed as a switch, activating the electronics and triggering then an alarm when high temperatures, thus entailing for possible fires, are detected.

Unlike smart bins, other components are embedded in the smart drop-off container architecture: an RFID reader and a GPS module. The RFID reader is a 125 kHz RFID card reader by Parallax, whose purpose is to detect and read the ID of users smart cards allowing the opening of the drop-off container whenever authorized users authenticate. The RFID reader is then always kept on: when an RFID card is positioned close the reader the ID code stored inside is detected and sent via LoRaWAN to the server. The identity of the user is checked and then, if authorized, the drop-off container opening information is transmitted back through the downlink channel. While this operation could be performed in one of the two downlink windows foreseen of transmissions made by Class A devices, the operation of the smart drop-off container as a Class B device assures additional downlink slots in case of user authentication delays on the server side.

The second additional component is an Ultimate GPS Breakout Board by Adafruit. This device allows to retrieve the GPS position of the drop-off container every time this is required. Indeed, the GPS board is only turned on when required by means of a MOSFET acting as switch: this function is mandatory since drop-off container position is expected to be required asynchronously, exploiting the Class B downlink slots, only when the drop-off container location could be changed. Indeed, GPS modules are characterized by relatively high power consumption, thus preventing from keeping them always on.

Concerning data transmission, the same LoRaWAN module employed for the smart bin (i.e., RFM95W by HopeRF) was integrated in smart drop-off containers, adopting the same activation technique based on the use of a MOSFET as a trigger to limit the overall energy withdrawal only when actually required. Moreover, the same transmission parameters of the nodes implemented within smart bins were exploited.

Similarly as for smart bins, a rough estimation of the overall cost of a smart drop-off container can be estimated analysing the costs of the single components. In this case, since the device is notably more complex than the smart bin, a higher value is accounted: however, plain drop-off containers too are more expensive in comparison with bins, and the ratio among their cost and the cost of the electronics is comparable. In particular, the cost of the electronics for the smart drop-off container is estimated in ∼87 €: despite such higher value, drop-off containers are less subject to vandalisms rather than bins, and the overall lifetime of these devices is expected to be notably longer.

#### 3.2.2. System Powering

Smart drop-off containers structure features a large number of components characterized by relatively high power consumption. In particular, both GPS board and RFID reader feature high current absorption levels (in the order of tens of mA). At the same time, Class B LoRaWAN devices are inherently more power hungry if compared with Class A ones. For this reason, a system based on the use of PV panels for energy harvesting was set up. Indeed, such a structure can be easily placed on the top of the drop-off containers where a large surface is present. While the required amount of power is lower if compared to the one of VSUs (that will be presented in Section 3.3), the proposed architecture is based on a 10 W PV panel charging 2 series 3.7 V 18650 Li-Ion batteries like the ones employed in smart bins: battery charge is managed by a Battery Management System (BMS), whose purpose is to protect batteries from possible damages during the charge-discharge process.

Since the various components of the system feature different supply voltages (i.e., 3.3 V and 5 V), the system also integrates 2 MCP 1700 voltage regulators in charge of level the voltage to the required values.

### 3.3. Video Surveillance Units

VSUs are employed to detect anomalous activities near bins or drop-off containers: these may include waste abandonment, incorrect uses, vandalism or presence of fire. As a proof of concept, VSUs integrated in the proposed system architecture are conceived to perform this last task. In order to detect the presence of fire, VSUs are based on a hardware architecture that is able to sustain a notably heavy computational load: indeed, fire detection is performed exploiting a set of software tools based on machine learning algorithms for image recognition. Due to these technical features, VSUs are not intrinsically low power: nevertheless, two architectures were envisaged to manage the units supply.

#### 3.3.1. Hardware Setup

**Figure 4:**
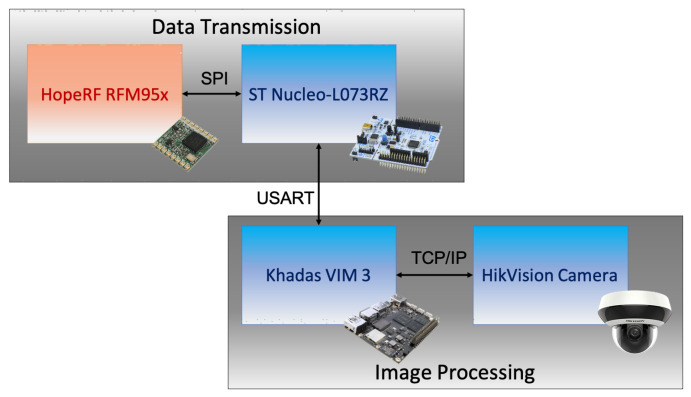
Video Surveillance Unit (VSU) block diagram.

The architecture of a VSU, whose block diagram is shown in Figure 4, can be divided in two subsystems: one is in charge of image capture and processing and the other is devoted to data transmission. The image processing subsystem is composed of an HikVison Mini PTZ Camera, that allows to capture single images that are retrieved by means of IP calls to the internal server of the camera, and of a processing unit. In order to reduce the complexity of the system, a Khadas VIM3 board was chosen: this device features an ARM64 Amlogic A311D processor, and it is also provided with an on-board 5.0 TOPS Neural Processing Unit (NPU) for the implementation of neural network operations. Image transmission from the camera to the VIM3 board is performed by means of a wired TCP/IP connection.

The data transmission sub-system is in charge of transmitting the results acquired by the image processing algorithms to the LoRaWAN server. This task is fulfilled by means of an Hope RF RFM95W LoRaWAN module (i.e., the same employed also for smart bins and smart drop-off containers) whose operation is managed through SPI connection by an STMicroelectronics Nucleo L073RZ board, which is connected to the Khadas VIM3 board via the Universal Serial Asynchronous Receiver Transmitter (USART) interface.

Due to the large number of costly components, the VSU is the most expensive of the three types of end nodes. Indeed, the overall cost of a single VSU can be estimated as ∼522 €: however, while such value is high compared to the other devices, it must be kept in mind that a single VSU can be exploited to monitor more devices. Moreover, VSUs can be placed in elevated places in order to avoid possible vandalisms and damages, thus ensuring a long lifetime.

Albeit VSUs may run under two different operating principles (i.e., synchronous and asynchronous), as it will be explained later on, the communication procedure amid the two boards stays the same and works as follows:

- As soon as the Nucleo L073RZ board activates, it requests data to the Khadas VIM3 via USART;
- The Khadas VIM3 board takes charge of the request triggering all the image processing routines devoted to spot fires within the pictures taken from the camera;
- Once that the image is analyzed, the Khadas VIM3 board arranges the response to the Nucleo L073RZ board request according to a predefined protocol thus sending the aforesaid response via USART;
- The Nucleo L073RZ board processes the incoming message and it accordingly broadcast a LoRaWAN packet (by making use of the same transmission parameters of the nodes implemented within smart bins) whose payload contains data related to the presence of fire in the nearby of the bins or drop-off containers.

In order to implement such serial communication as smooth as possible thus limiting re-transmissions with the aim of speeding up the procedure, the Nucleo L073RZ board manages USART peripheral according to the Direct Memory Access (DMA) paradigm. That is also the reason why this board was preferred in favour of simpler hardware solutions (e.g., an ATtiny84 as the one within smart bins) since the most of the latter ones are devoid of DMA controllers.

#### 3.3.2. Software Tools and Operating Principle

Since the main purpose of the VSU is to operate according to the edge computing principle, and thus performing image processing activities directly on-node, a set of software tools was selected and developed to perform the detection of specific assets or activities. In particular, as already anticipated, as a proof of concept, the VSU set up for the proposed framework was conceived with the purpose of detecting the presence of fire nearby bins and drop-off containers. The software infrastructure for the VSU exploits the following tools:

- You Only Look Once (YOLO): a machine learning tool customized for object recognition in images and video streaming;
- Yolo Fire Custom: a YOLO neural networking tool ad-hoc customized for fire detection [77];
- Server Manager: a software ad-hoc developed in Python programming language for the image acquisition from the HikVison Mini PTZ Camera, their processing and the subsequent transmission of the extracted information by means of the data transmission sub-system.

The whole architecture aims at performing the following operations: the Khadas VIM3 board is expected to process the images coming from the camera. The analysis will be carried out by the YOLO software having customized weights for fire detection. Once the presence of fire is detected in one of the processed images, a string communicating the alert will be transmitted by means of the LoRaWAN transmission channel. This procedure may be implemented either synchronously or asynchronously. In the first case, a duty-cycling procedure will be set up exploiting the Nucleo L073RZ board, which is the less power consuming component of the overall system. The Nucleo board will then activate according to the required sampling rate (e.g., once per hour), activating in turn the whole image processing sub-system: the information from the collected images will be then extracted and eventually transmitted to the server. This operative mode does not require any downlink transmission: for this reason, the data transmission sub-system will implement just a Class A LoRaWAN device.

Concerning the asynchronous functioning, this will be based on the use of the temperature data collected by the smart bins and the smart drop-off containers, that will be used as a preliminary alert. When the temperature value will exceed a pre-defined threshold, the server will transmit a downlink request to check for the possible presence of fire exploiting the VSU. In this case, the data transmission sub-system will implement a Class B LoRaWAN node and it will then trigger the image processing sub-system only when it will receive a request from the server.

#### 3.3.3. Power Management Strategy

Both the operating principles described in the previous section present pros and cons in terms of power consumption. Indeed, the synchronous modality requires the whole system to wake up every hour (or even less if required) and perform a full operating cycle. Similarly, the asynchronous mode requires the implementation of a Class B LoRaWAN device which requires the LoRa module to periodically wake up to set up the reception windows. In both cases, the powering of the VSU for prolonged periods relying only on batteries is technically unfeasible. For this reason, the only viable solution was envisaged in setting up an energy harvesting solution similar to the one already presented for the smart drop-off containers.

Nevertheless, energy requirements of VSUs are by far larger than the ones of smart drop-off containers if taking into account only the camera, according to the datasheet [78], its power consumption can be up to 8 W: even with a strict duty-cycling policy and with short operating periods, a set of batteries would last just a few weeks. In order to ensure self-sufficiency to the device, each VSU is then expected to be powered by an energy harvesting composed of the following items:

- 2 20 W PV panels;
- one 12 V 25 Ah lead-acid backup battery;
- one CMTD-A2420 solar charge controller, avoiding the battery to be damaged during the charge-discharge cycles.

This architecture was designed so as to operate according to the most energy consuming operating mode (i.e., the synchronous one).

### 3.4. LoRaWAN Network Architecture

As it was stated at the start of this section, the smart waste management system of Figure 1 is grounded on a LoRaWAN network whose nodes have been just introduced. Hence, within this subsection, the architectures of gateways and server side are introduced.

#### 3.4.1. Gateways

**Figure 5:**
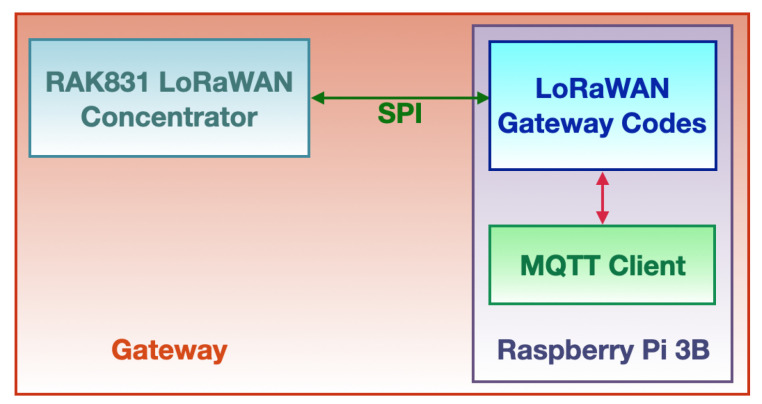
Gateway block diagram.

The gateways (i.e., LoRaWAN Class C devices) are in charge of demodulating the LoRaWAN packets and measuring either the Received Signal Strength Indicator (RSSI) and the Signal-to-Noise Ratio (SNR) for each of the incoming signals. Finally, they forward data and metadata of the packets to the cloud hosting the remote network server by exploiting the MQTT protocol. Concerning the gateway power supply, they are conceived to be mains powered. The gateway block diagram is shown in Figure 5.

The concentrator is a board embedding a LoRa modem and two LoRa transceivers. The RAK831 [79] produced by RAKWireless was chosen as concentrator: it is an 8-channel (i.e., the same channels on which the nodes transmit data) high performance concentrator which is capable of concurrently receive and demodulate up to 8 LoRa packets that could have been sent on as much channels even by exploiting different SFs. The RAK831 is composed by two SX1257 (i.e., LoRa transceivers) [80] and one SX1301 (i.e., a LoRa modem) [81], both produced by Semtech. Such an architecture allows for a sensitivity that varies according to the SF and the bandwidth: considering packets broadcast with a bandwidth of 125 kHz, the gateway sensitivity extends from −137 dBm at SF=12 to −126 dBm at SF=7. The computational burden the gateway is subject to is taken charge by a Raspberry Pi 3 model B [82] which also drives the concentrator via SPI protocol. Moreover, the Raspberry Pi runs all the needed software implementing the gateway routines and connects to the network server via the Internet over the MQTT protocol by resorting to a dedicated MQTT client that is implemented in a Python script. The software comprises a multi-thread program that demodulates the packets extracting the encrypted payloads and appending to each of them a series of metadata (e.g., RSSI and SNR). It is joined to the MQTT client which acts as a publisher connected to the MQTT broker running on the network server. The client conveys towards the broker all the data produced by the gateway multi-thread program by posting, on a gateway-specific MQTT topic, MQTT messages with a quality of service equal to 0 so as to shun message re-posting thus reducing latency. Similarly, the gateways transmit to the network server sundry functioning statistics (e.g., the number of received packets) on a periodic basis with a network diagnosis purpose. In order to enhance network performances by establishing a space diversity scheme, which is combined with the frequency diversity one that is implicitly introduced by the fact that nodes broadcast exploiting 8 channels, several gateways are deployed on the monitored environment.

#### 3.4.2. Server Side

**Figure 6:**
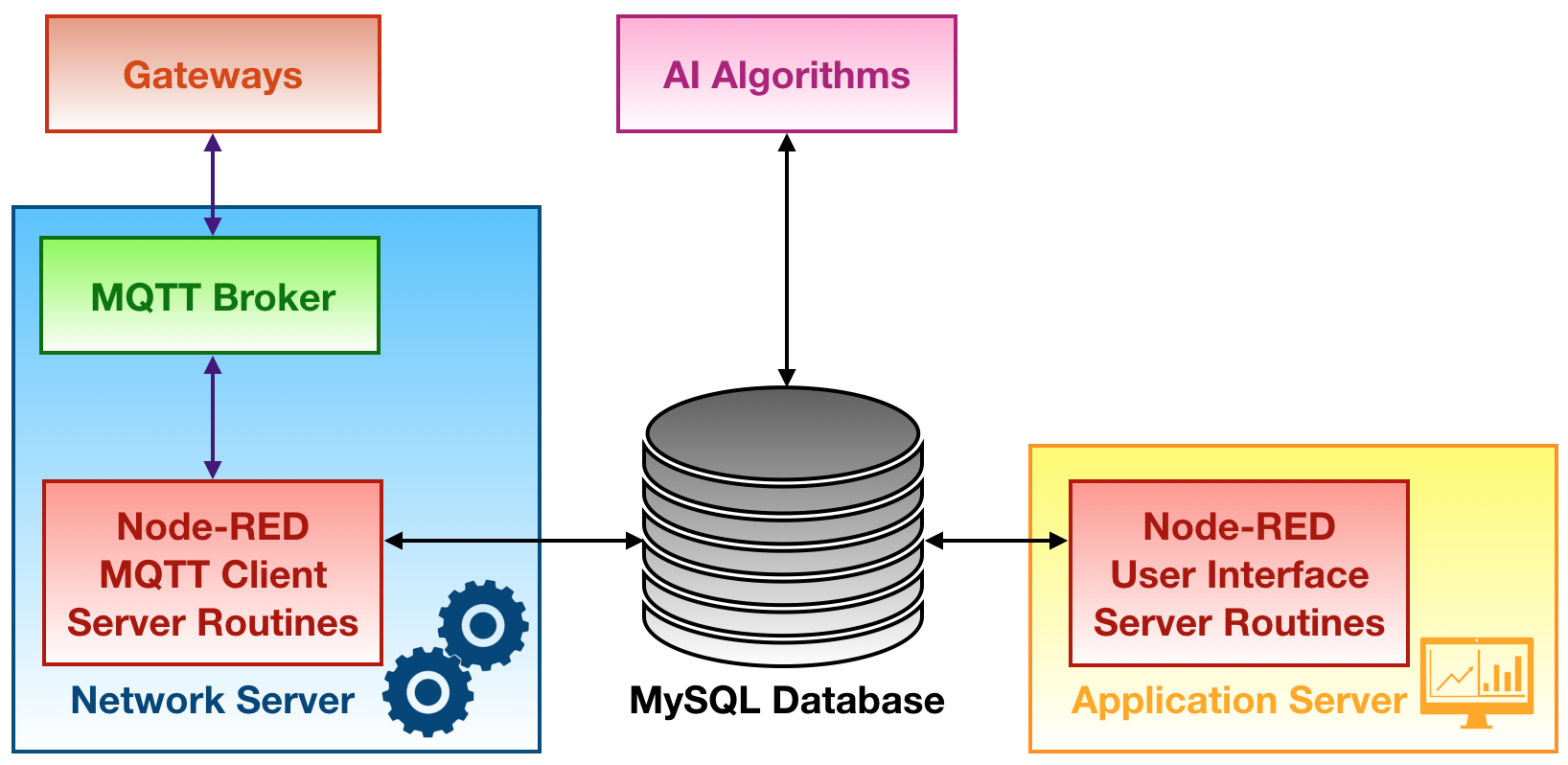
LoRaWAN server architecture block diagram. Violet arrows symbolize the MQTT connections while the black ones represent the connections to the database. Gateways and AI algorithms have been included just for the sake of completeness even though they are not part of the server side of the network: the former ones are scattered on the environment while the latter ones reside in the cloud too.

The server side of the LoRaWAN network is reported in Figure 6. It is mainly composed of a network server, an application server and a MySQL database that is assigned to the storage of all the data coming from end devices and gateways that are in turn processed by the network server. Both the software forming the network server and the application server are implemented by exploiting Node-RED [83]: a development environment created by IBM, which runs on Node.js [84] (i.e., a run-time environment executing JavaScript snippets outside a browser thus enabling their use for scripting on server side), that is based on a flow programming language whose building blocks are written in JavaScript.

The network server hosts an MQTT broker along with a Node-RED flow which carries out all the network server routines:

1. Send acknowledgement signals back to the gateways via MQTT, so as to fulfil network diagnostic, once that the MQTT clients running on the latter ones publish messages towards the client of the server;
2. Store the gateways functioning statistics in the database;
3. Filter out duplicated packets that may have been forwarded by some, or all, of the deployed gateways rejecting those with the worst RSSI;
4. Decrypt the payloads of the packets coming from the end devices by retrieving both the NwkSKey and the AppSKey which are previously stored in the database;
5. Discard the packets that could be sent by nodes belonging to diverse networks by performing the Message Integrity Code (MIC) check. This could happen since any end device that is included in other LoRaWAN networks encrypts payloads with different NwkSKey and AppSKey with respect to the ones stored in the MySQL database. Whenever their payload will be decrypted by making use of different AES keys (i.e., the one of the network of this smart waste management system), an invalid MIC will be experienced. Therefore, the network server will throw out those packets;
6. Store within the database the decrypted packets payloads, and their associated metadata, that passed the MIC check.

The application server is implemented by a Node-RED flow too: it extracts data related to either gateways and end devices from the database and displays them to users by means of a graphic interface containing widgets, plots and tables.

## 4. Test and Results

### 4.1. Preliminary Tests on Ultrasonic Sensor Accuracy

**Figure 7:**
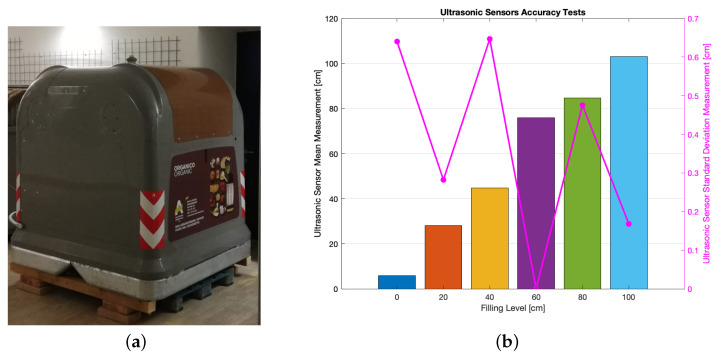
Ultrasonic sensor accuracy tests: (**a**) smart drop-off container indoor installed; (**b**) filling level trend throughout the various tests.

Since smart drop-off containers and smart bins share the same ultrasonic sensors to measure their filling level, such sensors underwent preliminary tests having as their purpose the assessment of their accuracy. Such trials were sorted out exploiting a smart drop-off container indoor installed within the laboratory (see Figure 7a): several filling levels (i.e., 0 cm, 20 cm, 40 cm, 60 cm, 80 cm and 100 cm) were reproduced within the drop-off container, then 200 measurements were performed, sent via LoRaWAN and stored in order to be analyzed (see Figure 7b). For each of the tested levels a cardboard sheet was put within the drop-off container at the level at hand, then several garbage bags were laid on it in order to simulate a real operation scenario. Higher filling levels were not tested due to the inability of sorting out a reliable garbage bags deployment for levels greater than 100 cm, because the drop-off container got smaller at that point thus making it barely impossible to enter within it so as to arrange garbage bags. Tests at 20 cm and 60 cm turned out to be less precise than the other ones. However, since the precise knowledge of the filling level was not crucial in contrast with the awareness of the occurrence of the event associated to the need of depletion caused by the maximum filling level, the ultrasonic sensor may be deemed to be reliable for understating the happening of the latter event.

**Figure 8:**
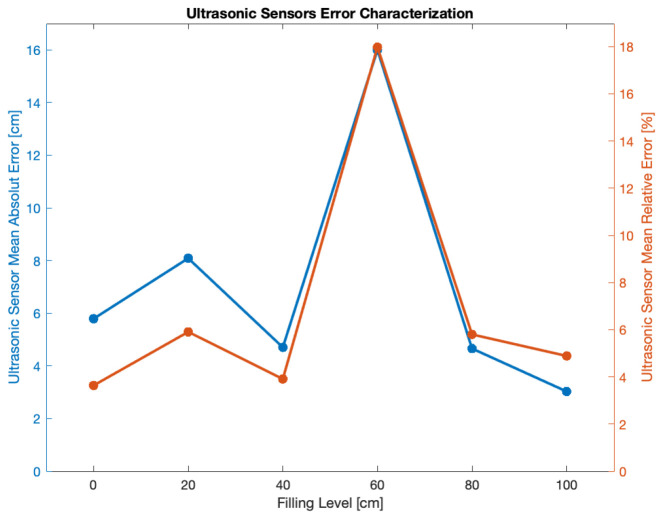
Absolute and relative errors of the measured values.

In particular, in Figure 7b mean values and standard deviations for the six measurement sets can be seen, while Figure 8 shows the absolute and relative errors. In general, the average absolute error for the six measurements was ∼7 cm; such a value was by far compliant with the assumption that the actual information that was expected to be obtained was empty, half-full or full evaluation rather than the exact filling level. In this sense, an error lower than 10 cm could be considered satisfying. Moreover, looking at Figure 7b it is possible to notice that standard deviation featured small values, thus suggesting a good level of reproducibility. Data shown in Figure 8 feature a peak for the filling level of 60 cm: in this case, the absolute error grew up to the value of ∼16 cm: at such a level however, a lower degree of accuracy was still acceptable since the actual crucial information concerned the full or empty status of the bin which was achieved for the 0 cm and 100 cm levels.

### 4.2. Smart Bins tests

**Figure 9:**
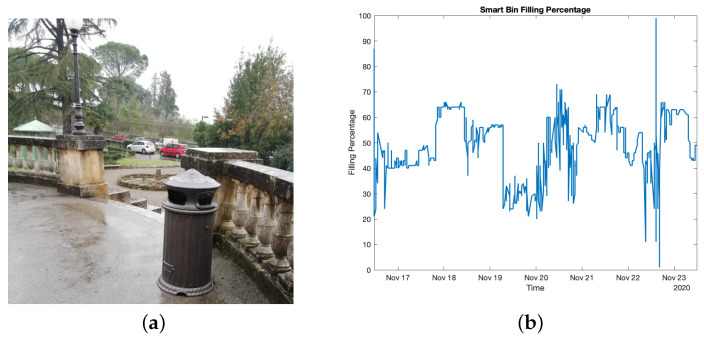
Smart bin test setup: (**a**) smart bin outdoor installed; (**b**) filling level percentage trend throughout the test week.

A smart bin was installed outdoors so as to test it throughout a week during which people used it as if it was installed in a real operation scenario (see Figure 9a). The objective of this test was to detect whether the bin was fully filled, rather than measuring the actual filling level, so as to promptly accomplish its depletion. Figure 9b shows the filling level trend, in percentage, throughout the test week.

At first glance, a high degree of fluctuation can be noticed. This is due to the fact that users threw heterogeneous rubbish. Indeed, suppose the bin is partially filled only with paper and then a heavier item is introduced thus causing a rubbish compaction. Despite such event causes a filling percentage reducing, it does not entail a depletion occurrence. This phenomenon could be observed several times during the test week, while only a depletion took place (i.e., almost at the end of the test period). Nevertheless, the important information for the public administration managing the waste collection operations was not the exact trash level but rather a rough indication of the bin filling percentage, and in this sense the system proved its effectiveness. Similarly, the most important information (i.e., the bin is full) was clearly visible. Likewise, it may be noticed that 0% filling was nearly never reached, meaning that the bin was never fully emptied. However, this was not exactly true because the 0% value was calculated at the actual bottom of the bin, while the the latter housed a garbage bag that increased this value of around 20% or even more whenever the garbage collector did not properly stretch the bag.

### 4.3. Smart Drop-Off Containers Tests

**Figure 10:**
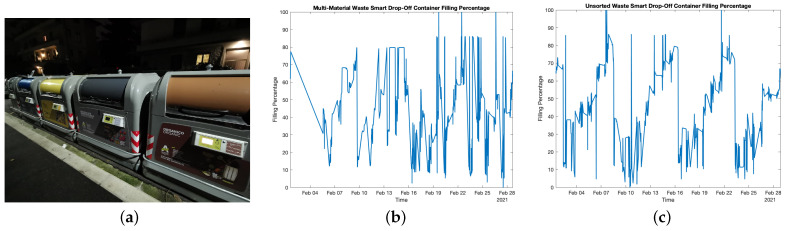
Smart drop-off container field tests: (**a**) smart drop-off container outdoor installed in the city of Florence, Italy; (**b**) filling level trend of multi-material waste smart drop-off container; (**c**) filling level trend of residual waste smart drop-off container.

These tests were along the same lines of smart bins ones: their aim was to detect whether the containers were fully filled, rather than precisely measuring their filling levels so as to trigger depletion procedures. Two smart drop-off containers were installed on the field in the city of Florence, Italy (see Figure 10a) and their filling level was sampled, sent via LoRaWAN and stored whenever users opened the relative lid after a successful authentication procedure via RFID. Such tests were carried out for a 4-week timespan, while the two smart drop-off containers were respectively devoted to the collection of multi-material waste (e.g., plastic and aluminium) and residual waste (e.g., whichever waste that cannot be disposed in other dedicated containers). Tests results are reported in turn in Figure 10b,c which show the filling level trend, in percentage, of the two smart drop-off containers.

Multi-material smart drop-off container was subject to 647 waste disposals and, in particular, at the beginning of the test period no disposals took place for 4 days after which the container was partially emptied since it was subject to a scheduled maintenance. Apart from this, the container was emptied five times and all of them happened within the second half of the testing period: such events can be noticed since the filling level trend shows five minima reaching values of approximately 2%. On the contrary, three maxima where the level reached 100% can be noticed: these phenomena occurred due to a momentary waste heap just below the sensor which was successively flattened after consecutive waste disposals. Similarly, this occurrence also happened several times during the test period even though no 100% filling level was recorded. However, these shortcomings did not hinder system effectiveness since such states were not prolonged meaning that no actual need of depletion is required.

Residual waste smart drop-off container was subject to 687 waste disposals, and it was emptied out five times: conversely from multi-material container, depletion procedures averagely took place every five days. Similarly as before, some maxima corresponding to 100% filling level, as well as slight filling level decreasing, occurred; the reason is the same as before since transient waste heaps formed that were dismantled with successive waste disposals. Once again, these weaknesses did not hamper system effectiveness for the same motivations explained earlier on.

### 4.4. VSU Tests

**Figure 11:**
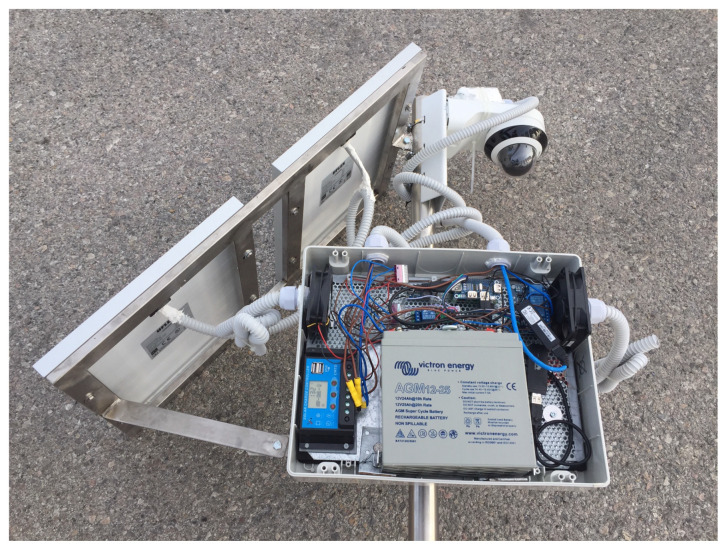
Prototype of VSU.

The VSU underwent two different set of tests. A first test was devoted to identify the hardware platform in charge of executing the image recognition software. For this purpose, and taking into account the containment of power consumption, two different boards were tested: a Raspberry Pi 4, characterized by lower costs and limited power consumption, and the Khadas VIM3 board, which was the chosen as the best solution. The first tests were made installing Tiny-YOLO, a lighter version of the YOLO software, on the Raspberry PI 4: while it was partially able to recognize objects, this approach was characterized by relatively long operating times. Indeed, depending on the image size, every image scanning generally required 5 ÷ 10 s, that is a relatively long time. Tests moved then on to the Khadas VIM3 board: in this case, the YOLO software was used. In these tests, the average scanning time was notably lower, below 1 s. Figure 11 shows the final prototype of the VSU together with the energy harvesting system.

**Figure 12:**
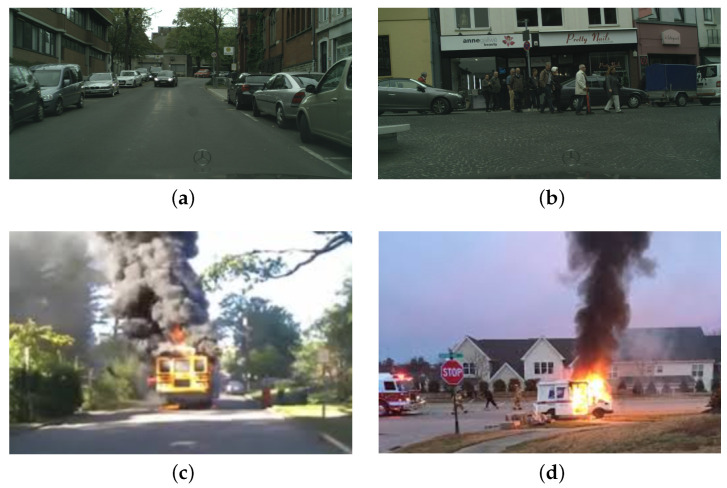
Examples of the images exploited for the assessment of VSU performances: (**a**) comes from [86] and represents a TN; (**b**) comes from [86] and represents a FP; (**c**) comes from [85] and represents a FN; (**d**) comes from [85] and represents a TP.

**Figure 13:**
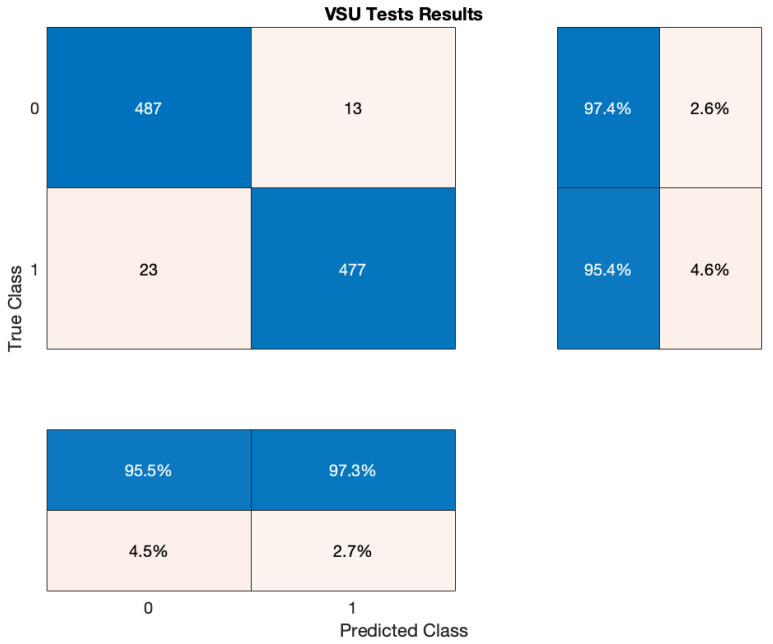
Confusion matrix relative to VSU tests.

Once we identified the ideal hardware platform, tests moved on to the second phase that was centred on the fire detection capability of the system. Due to the obvious inability of setting fires either in laboratory or within the field (i.e., the city centre of Florence, Italy), fire detection capability was proved by resorting to two online data-sets, each of which contains 500 pictures: for what concerns fire images, Ref. [85] was exploited; while regarding images that do not contain fire, Ref. [86] was selected since it includes pictures of urban areas so as to resemble to the actual test site. In particular, and with the aim of showing some of the pictures that were exploited for evaluating VSU performances, Figure 12a,b are extracted from [86], while Figure 12c,d are retrieved from [85]. Tests results are reported within the confusion matrix in Figure 13, where label 1 either in the predicted and true classes stands for the presence of fire, while the label 0 stands for its absence. Generally, 487 True Negatives (TNs) (e.g., Figure 12a) and 477 True Positives (TPs) (e.g., Figure 12d) were recognized, while 13 False Positives (FPs) (e.g., Figure 12b) and 23 False Negatives (FNs) (e.g., Figure 12c) were detected. Given this, VSU accuracy (ACC), False Discovery Rate (FDR) and False Omission Rate (FOR) can be defined as follows

(6) $$ACC=\frac{TP+TN}{TP+FP+TN+FN}100≃96.4\%,$$

(7) $$FDR=\frac{FP}{TP+FP}100≃2.7\%,$$

(8) $$FOR=\frac{FN}{TN+FN}100≃4.5\%.$$

According to the aforesaid tests, the VSU was potentially well designed so as to be directly employed in the field. Indeed, its ACC could be deemed as satisfactory and its FDR and FOR, even if they were limited, were not issues at all due to the fact the overall smart waste managements system also accounted for data coming from smart bins and smart drop-off containers. For instance, FP could be discarded since supposing the VSU recognized a fire, then temperatures measured by the bins or the containers would be low. On the other hand, FN may be tackled by the fact the bins or the container would sense elevated temperature. This additionally remarks the high level of data abstraction which was obtained by combining all of the data coming from the various nodes of the network.

## 5. Discussion

The results presented in the previous section demonstrate the effectiveness of the proposed multi-layer approach. Such an integrated approach is at our knowledge novel within the Smart Waste Management domain, where in general other traditional data transmission technologies like the cellular ones are employed when the complexity of the monitoring platforms goes beyond the transmission of data collected from single sensors. Conversely, the LoRaWAN-based approach proposed in this paper, applied to any kind of intelligent node in the network, including edge computing devices exploiting neural networking for image processing, can lead at the same time to a reduction in the overall infrastructure costs and to a simplification in the whole data management structure. In addition, the novelty of the system is highlighted by the fact that the entities composing the system are able to simultaneously accomplish the following tasks: measuring the filling level and the temperature of both the bins and the drop-off containers, detecting vandalism to both bins and drop-off containers, localizing drop-off containers position, authenticate users and performing video surveillance without harming people privacy. Moreover, the significance and the importance of the proposed network architecture proposed are both derived from the fact that such a system may be extremely useful for the acquisition of data coming from several activities, apart from waste management, falling within the context of Smart Cities (e.g., pollution monitoring). Therefore, this further highlights the architecture flexibility which is especially due to its multi-layered topology.

Of course, some problems and limitations did emerge during the tests phase. First of all, a relative level of inaccuracy was noticed for what concerns the filling level measurement systems, in particular for the case of bins. Indeed, in general ultrasonic sensors feature a limited dependence on temperature, that may be easily compensated via software. Nevertheless, this compensation was not carried out since the relative error is in the order of few cm and was assumed as not relevant for the filling level status. A second limitation that emerged concerns the usage of the sensors for the bins: indeed, in this case the measured level is subject to fluctuations that are mainly attributable to the narrowness of the bin itself, in comparison with the drop-off container (as it can be seen comparing Figure 9b with Figure 7b). Better performances in this case may be achieved by adopting a custom designed cap for the ultrasonic sensor [2]. At the same time, in the case of bins, level fluctuations are also due movements that occur at the disposed waste: for this reason, the identification of the percentage of filling (for example 0%, 50% and 100%) is more important than the exact level measurement in the case of bins.

Concerning the VSU, the most significant limitation that emerged during the tests was the impossibility to use a low-cost, general purpose single-board computer like Raspberry Pi, due to the unsatisfactory accuracy level of the image processing algorithms. As discussed in this paper, in order to achieve accurate results we were forced to adopt a platform characterized by an on-board NPU that was in charge of exclusively processing the AI-based image detection algorithms, with an increase in the cost of the devices and a larger power consumption.

Nevertheless, regardless of these drawbacks, the proposed approach, fully exploiting the features of LoRaWAN protocol, can be easily applied also to other application scenarios, within the Smart Cities paradigm as well as in other contexts. Within Smart Cities, such an infrastructure may be seen in particular as a backbone for all those services not relying on high bit-rates but requiring the deployment of a large number of intelligent devices in very wide areas.

## 6. Conclusions

The aim of this paper was to present the architecture of a Smart Waste Management infrastructure based on the LoRaWAN transmission technology, going beyond the single-purpose operation of a wide range of solutions that are currently found in the literature. For this purpose, three different platforms were joined together into the same data acquisition infrastructure, in order to manage all the trash disposal activities, being them carried out either at bins or drop-off containers, ensuring at the same time the security of the disposal sites by means of edge computing powered VSUs. All the growing levels of complexity of the developed prototypes, required the full exploitation of the different typologies of devices allowed by the LoRaWAN protocol, leading to a customization of the networking functions of each device according to its specific purposes. The resulting prototype was tested within a public area (i.e., a University Department) aiming at mimicking its future application scenario (i.e., outdoors on the roads). Moreover, the exploitation of each of the network layer enables to gain a higher level of data abstraction starting from simple information as the one acquired by smart public waste bins, smart drop-off containers and VSUs. Indeed, either at the middleware and at the application layer of the system, the combination of the aforesaid data gives rise to newer knowledge and contents that are handled at the business layer so as to optimally control all of the procedures entailed by the framework of waste management and disposal.

In conclusion, this paper tackles the problem of Smart Waste Management tempting to put forth a novel system. The latter can be exploited by municipalities for optimizing all the involved processes within waste management and disposal. Albeit for the time being the system beforehand presented is at its prototypical stage, it is able to monitor the filling level of both public waste bins and drop-off containers along with their state and whether they underwent vandalism. In addition, the employment of VSUs allows for fire recognition within Smart Cities paradigm. Finally, in the near future a redesign of the system is envisaged so as to proceed to field tests.

## Figures and Tables

**Table 1:** Functioning periods and currents draws for each firmware cycle for each of the smart bin components.

Component	Component	Functioning	Current
Name	Role	Period [s]	Draw [mA]
MCP1700	Voltage Regulator	$t_{1}=1800$	$c_{1}=0.0016$
ATtiny84	Microcontroller (Active Mode)	$t_{2}=5$	$c_{2}=0.490$
ATtiny84	Microcontroller (Sleep Mode)	$t_{3}=1795$	$c_{3}=0.0046$
RFM95W	LoRaWAN Module	$t_{4}=0.070$	$c_{4}=90$
JSN-SR04T	Ultrasonic Sensor	$t_{5}=3$	$c_{5}=8$
TMP36	Temperature Sensor	$t_{6}=0.020$	$c_{6}=0.050$

**Table 2:** ATtiny1604 microcontroller technical features.

Characteristic	Value
Maximum Clock Frequency	20 MHz
Flash	16 kB
SRAM	1024 B
EEPROM	256 B
Interfaces	USART, I2C, SPI
GPIO pins	12
ADC pins	10
